# Supplementary material for: Genetic Diagnosis of Retinoblastoma Using Aqueous Humour—Findings from an Extended Cohort
Source: Cancers (Basel). 2024 Apr 19;16(8):1565. doi: 10.3390/cancers16081565 (PMC11049382; doi:10.3390/cancers16081565)
Supplement: Supplementary file 1 [file cancers-16-01565-s001.zip › cancers-2920790-supplementary Figure S1 - AG edit.pdf]

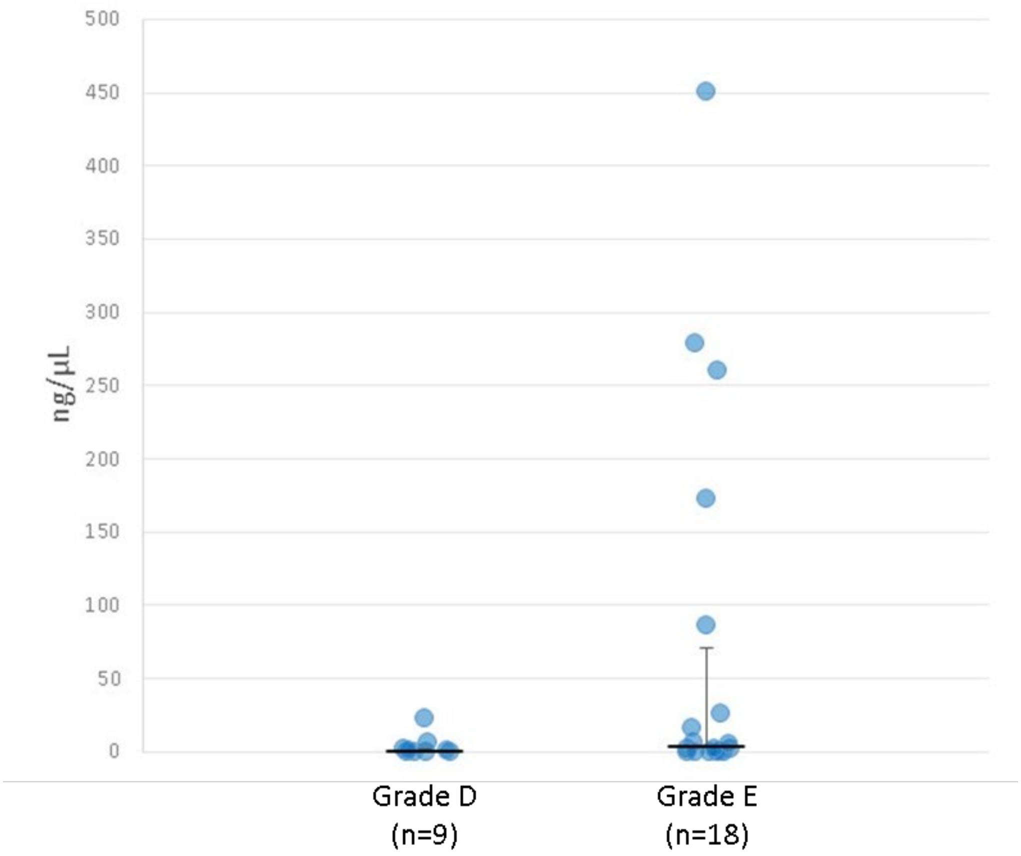

Figure S1. Cell-free DNA concentration of PE samples stratified by IIRC Grade. Median (black bar) and IQR are shown. The difference in cell-free DNA concentration between Grade D (0.35 ng/µL) and E (3.64 ng/µL) eyes, did not reach statistical significance ($p = 0.19$).
